# Future-Proof Regulation against the Test of Time: The Evolution of European Telecommunications Regulation

**DOI:** 10.1093/ojls/gqac016

**Published:** 2022-09-16

**Authors:** Pablo Ibáñez Colomo

**Affiliations:** London School of Economics and College of Europe

**Keywords:** economic regulation, administrative law, public law, EU law, Internet

## Abstract

Regulation is sometimes designed to be future-proof, so that it can adapt to changing economic and technological realities. The EU (and UK) Regulatory Framework for electronic communications was expressly crafted to be able to adjust to the evolution of the industry. This article considers how well the regime has stood the test of time and, based on this analysis, what lessons can be drawn for regulation more generally. It appears that, by and large, the Framework has effectively accompanied the transformation of telecommunications in Europe. On the other hand, the EU legislature’s commitment to future-proof intervention has waned over time. Every new review of the regime has represented a move away from the philosophy and mechanisms conceived to ensure that regulation would adapt seamlessly to industry shifts. This experience suggests that the failure or success of future-proof intervention primarily hinges on the intertemporal consistency of legislatures.

## 1. Introduction

Time—or, more precisely, the changes it brings about—is a source of anxiety for agencies and legislatures.^1^ Without the appropriate design, the regulatory framework that applies to a fast-moving industry may become obsolete, irrelevant or a source of distortions. A regime may turn out to be ineffective if technological shifts allow firms to circumvent the obligations to which they are (in theory) subject. Alternatively, it may create two or more tiers of firms—some subject to stringent duties, others escaping them for no reason other than the way in which its radius of action has been defined. Insofar as it does, the system in question may well regulate the industry, but may do so in unintended or fortuitous ways—that is, as a side effect of its inability to anticipate change and avoid unwarranted distortions.

The flaws of obsolescent regulation could in principle be addressed by introducing or amending legislation. The legislative process, however, is known to be a lengthy and unpredictable path. As a result, it may not be nimble enough to address change in an effective and timely manner. An alternative approach, on which this article focuses, is to craft regimes in a way that allows implementing agencies to seamlessly adapt and adjust any obligations to shifting economic and technological realities. The ambition under this second approach is to ensure, by design, that regulation is future-proof.^2^ Future-proofing legislation involves the use of techniques that introduce a degree of flexibility that is rarely found in the more traditional regimes. More precisely, it may rely on review clauses and/or may give regulatory authorities the power to modulate their responses to a changing landscape.

The EU (and UK) Regulatory Framework for electronic communications (hereinafter, the Framework)^3^ was expressly conceived as future-proof legislation. In fact, the very choice of words (‘electronic communications’, as opposed to ‘telecommunications’) is a manifestation of this ambition. The process of convergence^4^ between the audiovisual, IT and telecommunications industries advised in favour of the adoption of a regime that would adjust to the technological shifts that were well under way at the time. In particular, it was anticipated that some of the concerns justifying action in the wake of liberalisation would be organically addressed by the evolution of the industry alone. For instance, cable television operators had already emerged as rivals to incumbents at the time.^5^ In the late 1990s, when the Framework was proposed, it was understood that intervention could become redundant, if not counterproductive, over the long run.

The EU legislature crafted a regime that defined a set of principles and objectives to be achieved by regulatory authorities. As explained in section 2, it is for the agencies to evaluate, on a regular basis, whether intervention is justified in a given segment of the industry and, if so, to determine the most appropriate instrument to address any potential concerns. By decoupling objectives and instruments, and by leaving the choice of the latter to the authority, the EU legislature expected that the sector would be gradually deregulated with the progressive erosion of the incumbents’ positions in formerly monopolised markets. It was also expected that intervention would minimise its distortive effects by confining it to the segments in which it would be strictly necessary.

This article considers how well the Framework has stood the test of time, that is, to what extent it has proved to be genuinely future-proof. The point of the exercise is not only to examine the evolution of this particular regime, but also to draw lessons, more generally, for the design and operation of regulation. The conclusions are mixed. On the one hand, the basic design of the Framework has, by and large, achieved its initial goals and has proved able to adapt to the fundamental transformations of the industry. As explained in section 3, it seamlessly adjusted to the sweeping changes in the relevant economic and technological landscape. If at all, an analysis of the successive reviews of the Framework reveals some flaws, at the margin, in the regime. Some of these—addressed in section 4—relate to the definition of its substantive scope—such as the outright exclusion of audiovisual content.^6^ Other flaws concern the unclear legal status of some services (such as instant messaging and VoIP (voice over Internet protocol)) competing with those offered by incumbent operators.^7^

A critical evaluation of the successive reviews of the Framework, conducted in section 5, concludes that legislatures may be insufficiently committed to future-proof regulation. This is the key teaching to be drawn from the exercise and, arguably, is its fundamental contribution to the existing body of literature. Every single addition and/or amendment to the regime has invariably signalled a move away from the principles on which it was originally based. First, the decoupling of objectives and instruments, which was a hallmark of the Framework, has given way to more traditional regulatory techniques. Second, the reluctance to interfere with market structures and outcomes has been progressively abandoned in favour of a different philosophy, more confident about the ability of regulators to reshape industries.

The move away from the approaches, mechanisms and procedures originally enshrined in the Framework hints at several lessons for debates on regulation in other sectors. In particular, the evolution of the regime suggests that the key to the success of future-proof intervention and its survival is not the appropriate design of the legislation or the definition of its scope. Rather, the fundamental challenge is to ensure the commitment, over time, of legislatures to the principles originally underpinning regulation. These principles, conceived to preserve its adaptability, may not stand the test of time. The real threat to future-proof intervention, in other words, comes from the dynamic inconsistency that actors crafting and amending regulatory regimes are likely to display.

The evolution of the Framework suggests that the decoupling of objectives and instruments, as a regulatory technique, is likely to be short-lived. In the same vein, the initial impetus behind future-proof regulation may fade. Section 5 identifies several factors that might explain the declining commitment to flexible and adaptable approaches to intervention. One is the introduction of new regulatory objectives. A second one is the relationship between, and hierarchy of, aims over time: the prioritisation of some objectives over others may lead, in practice, to the use of different techniques. Finally, and more generally, the idea of future-proof regulation is premised on the perpetuation of a particular approach to intervention in the economy and understanding of the role of the state.

## 2. Principles of Future-Proof Regulation

### A. Background

In 1998, telecommunications activities were fully liberalised in the EU.^8^ The process that would lead to the elimination of exclusive and special rights in the industry was, to a significant extent, driven by technological change.^9^ In its 1987 Green Paper, the European Commission (hereinafter, the Commission) noted that incumbent operators, protected at the time by exclusive rights, would lack the ability and incentive to make the most of the opportunities opened by emerging services. In this sense, competition was deemed instrumental to achieving innovation, experimentation and flexibility.^10^ In the years that followed the publication of the Green Paper, the Commission’s efforts focused on dismantling the regime insulating incumbents from rivalry.

Once liberalisation was achieved, the Commission set out the principles for a new framework for the telecommunications sector.^11^ The ambition was to adopt a lasting regime. It was understood that its very design would be a challenge. The phenomenon of convergence between telecommunications, audiovisual and information technology services explained, by and large, the complexity and magnitude of the task.^12^ The progressive coming together of the three industries meant that some regulated activities were likely to undergo a fundamental shake-up in the subsequent years. It also meant that the overwhelmingly dominant positions of incumbent operators had become vulnerable (and this in ways that could not be fully foreseen at the time).

Suffice it to mention some examples to illustrate the impact of convergence. Convergence at the level of networks made it possible to use them interchangeably. The radio spectrum could be relied upon to provide not only audiovisual but also telecommunications services, thereby circumventing the segments of the legacy infrastructure with natural monopoly features.^13^ The same can be said of cable television networks.^14^ Conversely, the telecommunications infrastructure could be used to provide audiovisual content. Convergence had the potential to inject competition at the level of services, too. The Internet (a decentralised network that can be aptly described as a general-purpose technology^15^) made it possible to provide, *inter alia*, voice telephony and audiovisual content in competition with incumbents. In addition, it paved the way for innovation.

Against this background, the regulation of convergent activities had to be designed in a way that intervention would not become an obstacle to competition and innovation at the level of networks and services. In particular, if a network could be used to offer services in competition with the incumbent (say, a cable television network), regulation was not to stand in the way. Achieving this goal requires, in the first place, eliminating legal barriers to entry. It demands, in the second place, crafting regulation around neutral legal concepts that encompass substitutable infrastructure and services, irrespective of the underlying technology. It makes it necessary, in other words, to ground regulation on substance, as opposed to formal labels or legacy categories.

Convergence is a challenge for a second reason. Regulation is typically based on a series of assumptions about the technological and economic features of an industry. In rapidly moving sectors, just like telecommunications was in the late 1990s, some of these assumptions may become obsolete as structural shifts take place. For example, a key assumption in early regimes was that successful entry demands obligations mandating access to the incumbent’s infrastructure. This is so on account of the natural monopoly features of the last segment of the telecommunications network. This assumption, however, would not be accurate once technological and economic developments allow new entrants to bypass the incumbent’s local infrastructure (for instance by relying on wireless technologies). If regulation does not consider the potential for such evolution, it may perpetuate access obligations even when they are no longer required to promote and preserve competition (and may even be counterproductive).

The economic and technological evolution of a dynamic industry may raise challenges for a third reason. Just like regulation may assume that some concerns require permanent intervention, it may be unable to anticipate new and different market failures justifying action. In the wake of the liberalisation process, the issues requiring intervention were essentially two: access to the incumbent’s local infrastructure and interconnection between networks. As the sector evolves, however, unforeseen concerns may emerge. These may relate, for instance, to the launch of novel services, or to a shift towards oligopolistic market structures.^16^ Unless the regulatory framework is designed to respond to these new challenges, it will not be in a position to address them.

### B. Principles of the Framework

In its 1999 Review, the Commission sketched the principles of a future-proof regime. First, the document explained that regulation would be technologically neutral,^17^ and thus would not discriminate based on the technology underpinning a network or a service.^18^ Any choices would be left to market forces, rather than the regulator. The Framework adopted in 2002 achieved neutrality by developing legal concepts applying across the board to all activities. In fact, an emblematic aspect of the new legislation and its commitment to a future-proof approach to intervention is the replacement of the word ‘telecommunications’ by the words ‘electronic communications’. This symbolic move sought to reflect the idea that the legacy infrastructure and the incumbent operator compete with emerging networks.

Two technologically neutral concepts were introduced in the 2002 Framework: ‘electronic communications network’^19^ and ‘electronic communication service’.^20^ This approach reflects the vision of the 1999 Review, which identified three layers: at the bottom is the infrastructure (the ‘electronic communications network’^21^) over which services are provided; in the middle are the services immediately associated with the infrastructure (‘electronic communications services’^22^); and at the top are the activities provided via the electronic communications networks and services, such as audiovisual content and information society services. The upper layer was left outside the scope of the Framework, on the grounds that it is typically governed by other fields of law.^23^ This is obviously true as far as television content is concerned, which is subject to a strict sector-specific regime at the national and EU levels, but also of financial services.^24^

Second, the 1999 Review introduced a principle of minimal intervention. Accordingly, remedies would be confined to what would be strictly necessary to achieve the objectives of the Framework. By the same token, intervention would roll back once it appears that a market would be effectively competitive in its absence. There are three corollaries to this principle of minimal intervention. First, regulation was conceived to be of a temporary nature, in the sense that it would only come into play for as long as necessary. The principle demands that authorities review the need for action on a regular basis and withdraw remedies once a prospective analysis shows that it is no longer needed. A second corollary is that effective competition is deemed the best form of regulation and the ideal mechanism to attain the objectives of the regime in terms of, *inter alia*, price, choice or innovation. The third corollary is that regulation would not seek to influence market structures and outcomes. Subject to universal service provisions,^25^ the Framework, as originally conceived, left no room for industrial policy. Accordingly, it did not empower authorities to prescribe the services to be provided, the number of players in the industry, the degree of vertical integration or the levels of investment. These choices were left to market forces. The whole apparatus is indeed based on the idea that effective competition would also be the best form of industrial policy, and this insofar as it would spur firms’ incentives to invest and innovate.

Legislation embracing the principles of technology neutrality and minimal intervention needs to be calibrated to manage the trade-off between predictability and flexibility. On the one hand, legal certainty demands clarity about the instances in which obligations would be imposed. On the other hand, the future-proof nature of the regime demands an approach that makes it possible to adjust intervention to changing circumstances. The 1999 Review envisioned a regulatory technique that would not revolve around rules prescribing in detail the obligations to be followed by operators. Instead of laying down such duties, legislation would set out the objectives to be followed by authorities. It would be for the latter to craft the rules and ensure that they apply where necessary. In other words, the Review sketched a mechanism in which the objectives would be decoupled from the instruments used to achieve the said objectives.

The Framework, in its first incarnation, delineated the boundaries of intervention by regulatory authorities around three main sets of objectives. These were defined in Article 8 of the (original) Framework Directive (no longer in force).^26^ The first set of objectives was the promotion of competition and the maximisation of benefits for end-users in terms of choice, price and quality. The second was the development of the internal market. The third was the promotion of the interest of European citizens by means of, in particular, consumer protection and access to a universal service. The decoupling between objectives and instruments and, more generally, the operation of the Framework becomes particularly apparent in relation to the implementation of the first set of objectives, which revolves around the market analysis procedure described hereinafter.

## 3. Future-Proof Regulation in Practice

### A. The Market Analysis Procedure as the Ideal of Future-Proof Regulation

The market analysis procedure represents an ideal of future-proof intervention from both a substantive and a temporal standpoint. It is an apparatus designed to ensure that intervention remains consistent, over time, with the principles and objectives of the Framework. In its current version, the procedure is enshrined in Articles 63–82 of the EU Electronic Communications Code (hereinafter, the Code).^27^ These provisions define the instances in which intervention is warranted under the regime and the conditions that regulatory authorities need to satisfy before imposing any obligations. They also set out the steps to follow to review and, if necessary, roll back such obligations. From a temporal standpoint, the evaluation of the conditions of competition—and thus of whether intervention is, or continues to be, warranted—is to be undertaken, as a matter of principle, every five years.^28^

The market analysis procedure relies on competition law concepts.^29^ In particular, it demands from authorities the sort of context-specific evaluation that is characteristic of that field of law. Thus, there are two conditions for regulatory intervention, to be established on a market-by-market basis, under the Framework. First, an authority would need to show that an operator, individually or jointly with others, enjoys a position of significant market power.^30^ The notion of significant market power is defined directly by reference to the concept of dominance as understood in the context of article 102 TFEU.^31^ Accordingly, establishing such a position demands the definition of the relevant market^32^ and evaluating whether an operator (or group of operators) has the ‘power to behave to an appreciable extent independently of competitors, customers and ultimately consumers’.^33^

Under the Framework, a finding of significant market power is insufficient, in and of itself, to impose remedies. The letter of the Code makes it clear that regulatory intervention is only warranted where the relevant market has structural features that make it difficult for effective competition to emerge or be sustained. Whether a market presents such features is evaluated against the so-called ‘three-criteria’ test, currently enshrined in article 67(1).^34^ In accordance with the first criterion, intervention is only warranted on markets that display ‘high and non-transitory structural, legal or regulatory barriers to entry’. The second criterion provides that intervention can only take place on segments which do ‘not tend towards effective competition within the relevant time horizon’. This condition demands, in essence, a prospective analysis of the likely evolution of the relevant market. Finally, ‘competition law alone’ must be ‘insufficient to adequately address the identified market failure(s)’.

Pursuant to the Code, there are two ways in which the three criteria can be shown to be met. To begin with, there are markets that presumptively meet all three criteria. These are identified in a Recommendation that the Commission is required to issue (and to review on a regular basis to account for economic and technological developments) under the Framework.^35^ Second, a regulatory authority may be able to show, on the back of an individual assessment, that a particular market that is not identified in the Recommendation nevertheless meets all three criteria.^36^ The conditions that the agency would need to establish to discharge its burden are set out in article 67(2) of the Code. The successive versions of the Recommendation expressly caution against regulating emerging markets.^37^

Where the authority has established a position of significant market power on a segment that fulfils the ‘three-criteria’ test, it can consider the imposition of regulatory obligations. In line with the principles described above, the Framework does not prescribe the nature of the obligations to be imposed. Instead, it provides for a menu of options from which authorities may choose depending on the nature of the concern addressed. Pursuant to the principle of minimal intervention, regulators would not have the discretion to select the remedy in any given instance. Rather, the remedy must find the ‘least intrusive way’ to attain the objective.^38^ What is more, some obligations are subject to stricter conditions and can only be imposed on an ‘exceptional basis’.^39^

The remedies found in articles 69–74 and 76–80 of the Code range from the least to the most intrusive. At one end of the spectrum, one can identify obligations of transparency,^40^ non-discrimination^41^ and accounting separation.^42^ At the other end, there are remedies providing for the separation—functional^43^ or (where voluntarily proposed by the firm) structural^44^—of an operator. In between, one recognises the sort of obligations that are typically found in utilities regulation, which include price controls and access duties.^45^ Where necessary, these remedies may be combined: for instance, an access obligation may be imposed together with terms and conditions that might include price controls and a non-discrimination obligation.

### B. The Implementation of the Future-Proof Regime

The practical implementation of the market analysis procedure is testimony to its ability to adapt to the economic and technological evolution of the industry. To the extent that this is the case, it can be said to be a successful model of future-proof intervention. An analysis of the actual operation of the regime also shows how the principles of minimal intervention and technology neutrality have been put into effect. As the logic of the Framework would have predicted, the scope of intervention has rolled back as effective competition has gained ground in the sector.^46^ It appears, in addition, that the market analysis procedure has adjusted seamlessly to account for major transformations, namely the upgrade of incumbents’ networks and the deployment of alternative infrastructures. Third, the Framework has given national regulatory authorities the necessary leeway to experiment, on a routine basis, with remedies and procedures.^47^ These experiments, subsequently codified in legislation, have made intervention more flexible, effective and tailored to the features of specific markets.

The best way to exemplify how the scope of intervention regulation has shrunk as activities have become more effectively competitive is to compare the versions of the Recommendation on relevant markets issued by the Commission over the years. In the 2003 iteration, the authority identified 18 markets presumptively meeting the ‘three-criteria’ test.^48^ These markets were reduced to seven in the 2007 incarnation of the Recommendation^49^ and to four in 2014.^50^ The version issued in 2020 singles out a mere two markets.^51^ This trend reflects a decline in the range of activities susceptible to *ex ante* intervention. By and large, the remaining challenges for regulatory authorities revolve around access to the local telecommunications infrastructure.^52^

The local telecommunications infrastructure presents the sort of ‘high and non-transitory barriers to entry’ that new entrants struggle to overcome.^53^ This segment is still presumed to be the main remaining obstacle to ‘infrastructure-based’ competition—that is, a market configuration in which operators rely exclusively on their proprietary network to provide their services.^54^ Thanks to the flexibility afforded by the Framework, remedial action in this context has seen significant experimentation and innovation by authorities, ranging from the procedural (including the introduction of a commitments procedure^55^) to the substantive (including the concept of functional separation^56^). Similarly, and in accordance with the principle of minimal intervention, access obligations may no longer relate to the telecommunications infrastructure itself but to the civil engineering works through which the infrastructure is deployed.^57^

In retrospect, it appears that the single most remarkable quality of the market analysis procedure is its ability to adapt to a changing technological and economic landscape. Since 2002, the telecommunications sector has witnessed major transformations. First came the emergence of cable television operators as providers of broadband Internet services^58^ and, second, the large-scale deployment of fibre (and the progressive phasing out of copper infrastructure), which took place under competitive conditions and which required substantial investments.^59^ The third transformation, currently underway, relates to the dramatic increase in the speed, reliability and performance of mobile Internet, which might become an effective substitute for fixed broadband services in the medium to long run.^60^

The Commission, as much as national regulatory authorities, has remained remarkably committed to the principle of technology neutrality in spite of these transformations. As a result, intervention has adapted seamlessly to major shifts in the industry. The market analysis procedure has consistently applied in a technologically neutral way. For instance, the Commission has consistently rejected claims that upgraded infrastructure should be exempt from regulatory duties.^61^ Under the future-proof logic of the Framework, the fundamental question is instead whether two services (say, one provided via fibre and the other provided via copper) can be seen as substitutes from a supply and demand perspective.^62^ In practice, the access obligations that were imposed on the incumbent operator in relation to its copper infrastructure have seamlessly extended to the upgraded infrastructure, thereby securing the achievement of the objectives of the regime over time.

On the other hand (and this is the second key to its success as a future-proof regime), the Framework provides the necessary flexibility to modulate and refine regulatory duties depending on the specific circumstances of the relevant market. For instance, wholesale access charges to the incumbents’ infrastructure have been adjusted, if not deregulated altogether, over time.^63^ In the early days of the Framework, the typical remedy package in relation to the legacy copper infrastructure involved price controls in some form and was therefore relatively intrusive and far-reaching. By contrast, intervention concerning upgraded fibre networks is generally light-touch and may not impose anything other than a non-discrimination obligation to ensure that its own retail arm and competing ones are placed on a level playing field. The level of charges (and thus the reward for the firms’ investments) would fall outside the reach of intervention.

## 4. The Limits of Future-Proof Regulation

### A. The Limits of Legal Concepts

In spite of the successes outlined in the preceding section, the Framework as originally designed did not prove to be entirely future-proof. It appears, in hindsight, that there are two aspects that were not legislated in a manner consistent with its declared ambition. As a result, it has been necessary to amend the regime or to resort to other fields of law to fully capture the economic and technological reality to which it applies. First, the Framework excluded, outright, audiovisual services from its scope. This substantive choice was at odds with the reality and competitive dynamics of the industry. The resulting mismatch between regulation and reality has been imperfectly addressed through competition law. Second, the rise of ‘over-the-top’ (OTT) services such as Skype or WhatsApp, which compete with legacy offers, led to the redefinition of the substantive scope of the Framework. These two aspects are considered in turn.

Originally, the exclusion of audiovisual services from the Framework was justified on grounds that the activity is subject to another regime with different goals. The underlying assumption was that it is possible to draw a neat distinction between content-related and transmission-related activities. It did not take long for the flaws underpinning this assumption to become apparent. Here are some examples. A subscription to cable television involves both the transmission of signals and the provision of audiovisual content.^64^ Artificial as it may seem (and to the extent that the two can be meaningfully distinguished from one another), only the former is subject to the regime.^65^ Similarly, the licensing and the acquisition of the rights to audiovisual content do not qualify as ‘electronic communications services’. It is unquestionable, however, that such rights drive competition in markets subject to the Framework. Operators seek to attract consumers with offers combining content and broadband Internet access and gain a competitive advantage by providing premium offers on an exclusive basis.^66^ Again, these moves fall outside the scope of the Framework in spite of their undeniable impact on regulated activities.

Competition law has been the primary vehicle through which these gaps in the Framework have been filled. It is through that discipline (and, in particular, merger control) that authorities have sought to minimise the potential anticompetitive effects resulting from the integration of telecommunications operators and content providers.^67^ The enforcement of competition law has led to the imposition of obligations mimicking those mandated under the Framework. Thus, the clearance of transactions has been made conditional on incumbents providing access to content under regulated terms and conditions.^68^ Competition law is, in any event, an imperfect substitute for intervention under the Framework and fills gaps only partially, if at all. This is so not only due to the inherent limitations of competition law institutions,^69^ but also because the point of the discipline is not to erode positions of significant market power (as is true of the Framework) but to prevent their creation and/or strengthening.^70^

Inevitably, some of the responses to the impact of audiovisual content on regulated activities have proved to be inconsistent with the objectives of the Framework and/or with the logic underpinning market analysis procedure. In some cases, the introduction of *ad hoc* regulation filling the gaps of the regime has had the unintended effect of strengthening the incumbent and weakening new entrants’ positions. The Pay-TV consultation run by the UK regulatory authority, which led to the imposition of regulatory obligations on a new entrant, is a case in point.^71^ Instead of evaluating whether the markets for the exploitation of exclusive content were comparable to the markets satisfying the ‘three-criteria’ test, and instead of considering whether intervention was consistent with the overall objective of promoting effective competition in electronic communications markets, the UK authority in effect neutralised the new entrant’s competitive advantage *vis-à-vis* the incumbent.

The regulation of OTT activities is one of the factors that led to the amendment, in 2018, of the notion of ‘electronic communications service’. The purpose of this amendment was to make it clear beyond doubt that OTT services serving the same purpose as traditional ones are subject to the regime.^72^ Providers such as Skype or WhatsApp do not have a major impact on the objectives of the Framework. In fact, their entry is one of the outcomes sought by it, as well as the very expression of technological convergence. However, their outright exclusion from the scope of the Code would have been a source of distortions. This is so to the extent that electronic communications services are subject to some regulatory duties—including in relation to interoperability^73^ and emergency services.^74^ As a result, leaving them outside of the Framework would have favoured new entrants for no reason other than the arbitrary definition of the boundaries of the regime. What is more, such an outcome would be inconsistent with the principle of technology neutrality.

The review of the Framework that would lead to the adoption of the Code introduced a new definition of ‘electronic communications services’ that removed any ambiguity about the status of OTT activities. More precisely, article 2(4) of the Code makes it clear that an ‘interpersonal communications service’ qualifies as an ‘electronic communications service’ within the meaning of the Framework. That concept, in turn, is further subdivided between ‘number-based’^75^ and ‘number-independent’ services (the latter including, *inter alia*, WhatsApp).^76^ This approach allowed the legislature to modulate the regulatory obligations to which operators would be subject. In particular, it seeks to ensure that lighter duties are imposed on the latter category.^77^

### B. The Intertemporal Inconsistency of the Legislature

If there is one lesson to draw from the Framework and its evolution, it is that the limits of future-proof regulation do not come primarily from the flaws of regimes as originally designed. If European telecommunications regulation has become less future-proof over time, this is so, by and large, due to the tendency of the legislature to abandon the principles around which the regulation was initially crafted. With every new revision of the Framework, the legislature has displayed an inclination to depart from the market analysis procedure and the logic underpinning it. Thus, the decoupling of objectives and instruments has been progressively abandoned. As a result, intervention does not always rely on the mechanisms expressly conceived to ensure that it would be future-proof. Three key developments reflect the move away from the principles of minimal intervention and technology neutrality (and, by extension, from future-proof intervention): first, the regulation of roaming, which now revolves around *ad hoc* legislation directly setting the level of tariffs; second, net neutrality, which is subject to a *sui generis* approach; and finally, interconnection tariffs, which are no longer set in accordance with the market analysis procedure.

The regulation of roaming^78^ is arguably the issue that has attracted the greatest interest from non-specialist audiences. In 2007, the EU legislature chose to cap directly the charges that could be imposed on end-users travelling across borders within the Union.^79^ However popular, the measure is inconsistent with the market analysis procedure and the rationale underpinning the Framework. In accordance with the latter, intervention would be contingent on whether, on the relevant market(s) for roaming, a position of significant market power (by a single operator, or jointly with others) can be established. In the absence of such a position—and in line with the principle of minimal intervention—any roaming charges would be considered to be an expression of the prices and practices prevailing in an effectively competitive market (and this even when they do not coincide with the level of prices preferred by agencies and other public authorities).

The background of the original Roaming Regulation shows that the legislature departed from the principles of the Framework once the default approach failed to provide the outcome sought by authorities. Prior to the adoption of *ad hoc* legislation, the market analysis procedure was in fact followed. However, it led to the conclusion that no firm enjoyed a position of significant market power. Thus, price regulation of roaming was not deemed warranted under the market analysis procedure.^80^ In addition, the Commission opened an investigation under its competition law powers, but these proceedings were closed without a finding of infringement.^81^ Despite the absence of evidence of anticompetitive conduct and/or of significant market power, intervention took place, and roaming charges were eventually capped in legislation before being removed altogether in 2015.^82^

The 2015 Regulation that saw the elimination of roaming charges is the same that introduced strict net neutrality obligations at the EU level. The principle of net neutrality amounts, in essence, to requiring that the traffic flowing over the Internet be treated in a non-discriminatory manner by network operators.^83^ The fundamental goal of this principle is the preservation of the Internet as originally conceived, that is, as an ecosystem in which ‘intelligence’ is placed at the edges of the network and in which the underlying infrastructure fulfils a passive, or ‘dumb’, role.^84^ Under net neutrality, operators running the infrastructure are thus precluded from favouring, or discriminating against, some traffic. In practice, the non-discrimination principle encompasses practices such as blocking, slowing down or degrading the quality of content offered via the Internet.^85^

Whether or not intervention in the name of net neutrality is appropriate could be examined under the market analysis procedure. The question, under this approach, would revolve around the status of Internet access providers. Accordingly, remedial action would be warranted where they enjoy, individually or jointly, a position of significant market power *vis-à-vis* firms offering content over the Internet.^86^ Conversely, it would not be appropriate in an effectively competitive market featuring intense rivalry among access providers.^87^ In accordance with the principle of minimal intervention, it is not for regulatory authorities to interfere with the management of Internet traffic once positions of significant market power are eliminated. In an effectively competitive market, the conduct of individual operators is beyond reproach, even if it is at odds with the logic of the Internet as originally conceived.

Initially, the EU approach to net neutrality-related concerns was consistent with the market analysis procedure and the principles underpinning the Framework. In particular, the Commission advanced the idea that any preoccupations relating to net neutrality were, to the extent that they existed, the consequence of the asymmetry of information between Internet service providers and end-users. Similarly, the Commission expressed the view that any such concerns could be addressed by means of obligations specifying the nature of the service offered and, if necessary, with minimum quality requirements.^88^ That was also the initial response of the EU legislature in its 2009 review of the Framework.^89^ Eventually, however, fully fledged net neutrality duties expressly banning certain acts were directly introduced in legislation.

The final example that marks the departure from the principles originally informing the Framework is that of interconnection tariffs. From the outset, the termination of calls was included by the Commission among the markets susceptible of *ex ante* intervention under the market analysis procedure.^90^ The inclusion of these markets was based on the premise that each operator enjoys a position of significant market power in relation to the calls terminated in its own network.^91^ Accordingly, regulatory authorities around the EU imposed remedies to operators in the form of price controls.^92^ Article 75 of the Code abandoned this approach. Instead of following the market analysis procedure, the provision requires the Commission to issue delegated legislation directly fixing a single set of termination rates covering the EU as a whole.^93^

## 5. Making Sense of the Demise of Future-Proof Regulation

### A. Intertemporal Commitment and Regulatory Fragmentation

The discussion in the preceding sections shows that the Framework has paradoxically become less future-proof with time. The decoupling of objectives and instruments, which was originally at the heart of the regime, has been progressively abandoned by the legislature. The most pressing challenges in the industry are no longer addressed by means of the market analysis procedure. Several factors can explain, individually and jointly, this phenomenon. The first and arguably most relevant one is the inability of the legislature to commit to, and accept, the necessary consequences of the regulatory choices made at the outset. In other words, the observed intertemporal inconsistency seems to be, at least in part, a manifestation of the legislature’s inability to commit, over the long run, to an approach that requires accepting some outcomes that are not always palatable in the short term.^94^ As a result, future-proof regulation is inherently fragile, in the sense that it is contingent on the ability of institutions to credibly accept the choices made and their inescapable implications.

There are several reasons why the short-run outcomes resulting from the principles of minimal intervention and technology neutrality may be undesirable, even untenable, in practice. To begin with, regulation that relies on effective competition is unlikely to display its positive effects immediately. For instance, the low prices and the increased choice and quality that typically come with competitive markets are not manifested overnight. Second, the changes in market structures that result from effective competition involve trade-offs that are not necessarily acceptable or understood by stakeholders and/or legislatures. For instance, the desired balance between static (short-term) and dynamic (long-term) competition^95^ may be resisted; or its benefits may not be immediately grasped.

Promoting dynamic competition—that is, preserving firms’ incentives to invest and innovate. and ensuring they rival one another by improving existing products and developing new ones—is likely to demand sacrifices in terms of static competition (which may take the form of, *inter alia*, a short-term reduction in the choice of products or an increase in prices). Prices may rise, for instance, when the telecoms infrastructure is upgraded to fibre. Similarly, rolling back some regulatory duties may lead to the (temporary) departure of some operators (with the corresponding decrease in consumer choice). While these negative (static) effects may have an immediate, appreciable impact, the expected (dynamic) outcomes (in terms of increased innovation and investments) may take longer to display their positive consequences (or may be assumed as given without involving any trade-offs).

The regulation of roaming and net neutrality shows how difficult it may be for legislatures and regulators to commit to dynamic competition at the expense of static rivalry. Roaming charges, in the relatively early stages of the liberalisation process, are likely to be high (and significantly above cost). This fact, however, is not necessarily the consequence of a market failure or of a dysfunctional structure. High roaming charges are consistent with an effectively competitive market and as such are not necessarily undesirable. They may be borne by the relatively well-off end-users.^96^ In the same vein, a decision to eliminate roaming charges outside the market analysis procedure is likely to negatively impact those who are less well-off (who would, in effect, be subsidising wealthier customers). Just like price discrimination and cross-subsidies, relatively high margins on premium services are hardly unusual, and not necessarily a sign of lack of effective competition.

What is more, relatively high charges for roaming services need not be a stable state of affairs. To the extent that roaming markets are effectively competitive, demand for *ad hoc* services can lead to the adoption of tailored offers and bundles intended for frequent travellers.^97^ Roaming charges may also fall following the integration of operators across borders. Such organic market adaptations would provide a response that is consistent with the principle of minimal intervention (which would have advised against directly regulating roaming charges). From a political perspective, however, the short-term effects of high prices may make it unsustainable for regulators and legislatures to trust (and wait for) market forces to deliver. In addition, organic market adaptations may not necessarily benefit all consumers (or may disproportionately benefit relatively well-off users).^98^

Similar conclusions follow when one considers the issue of net neutrality. The ideal of neutrality is embraced to prevent practices favouring a firm’s affiliate or a particular third party at the expense of other third parties. For instance, intervention in the name of net neutrality would seek to prohibit a mobile phone provider from blocking a competing OTT service, such as WhatsApp. Similarly, it would preclude an Internet service provider from concluding an agreement with a content provider, such as Netflix, whereby the latter would benefit from preferential treatment over the network (in the form, for example, of zero-rating,^99^ or in the form of a ‘fast lane’^100^). Such arrangements would be at odds with net neutrality. However, they are hardly unusual in effectively competitive markets. In fact, vertical integration and exclusivity agreements are known to be a source of pro-competitive effects^101^ and can lead, *inter alia*, to the emergence of new and better services.^102^

The very practices that are problematic from a net neutrality perspective are healthy expressions of effectively competitive markets. Absent a finding of significant market power, there is no support, under the principle of minimal intervention, for a pre-emptive ban, across the board, of vertical integration, exclusivity agreements and other practices having an equivalent object and/or effect. These practices are routinely examined by competition authorities,^103^ and a careful case-by-case evaluation (as opposed to a blanket ban) has long been deemed appropriate for them. The same is true of the subsidisation of content providers (which is an inevitable economic effect of net neutrality). In an effectively competitive market, there are no obvious reasons to justify the transfer of rents from one level of the value chain to the other.^104^ What is more, subsidisation is not easy to reconcile with the principle of technology neutrality, which would leave choices concerning the allocation of rents within the value chain to market forces.

Even though the above is well known, it may be difficult for legislatures to remain committed to the principles underpinning the Framework. This is so for two interrelated reasons. First, the departure from the ideals of net neutrality may display acute static effects, namely the blocking or slowing down of certain services. Such effects may not be politically palatable and may lead to a short-term response.^105^ Second, in multi-level systems like the EU, there is a risk that legislative responses will lead to regulatory fragmentation within the internal market. If Member States perceive that action to address a departure from net neutrality is insufficiently strong, they may decide to enact legislation at the domestic level. In such circumstances, the EU legislature may opt for preventing regulatory fragmentation even when it comes at the price of undermining the principles of the Framework.

### B. The Recalibration of the Objectives of the Framework

There is a second reason why the Framework has become less future-proof: the objectives of the regime have been recalibrated over the years. As originally designed, the Framework prioritised the promotion of effective competition over other objectives. This choice explains why the market analysis procedure was at the heart of its operation. Other objectives, namely the development of the internal market and the protection of citizens’ interests, were subordinate to it. As already explained, the Framework was based on the idea that competition is the best form of regulation and, by extension, the most effective way to protect consumers and integrate national markets. The integration of Member States’ economies was to be attained, according to the logic of the Framework, by removing obstacles to market entry and by encouraging the development of trans-European networks and the development of the interoperability of services.^106^ Similarly, the creation of a universal service was achieved in a manner that would minimise any distortions of competition and would confine intervention to the areas where compensation would be needed.^107^

With subsequent amendments of the Framework, however, a rebalancing of the objectives took place. The development of the internal market, for instance, was not simply treated as a goal that would naturally come as a side effect of the promotion of effective competition, but as one that demanded positive measures aimed at integrating Member States’ economies. Roaming regulation, for instance, can be interpreted in this light. The eventual elimination of roaming charges sought to achieve market integration (and the harmonisation of national regimes) in a manner that contradicted the market analysis procedure but that led to immediate, concrete benefits for citizens. The same can be said of the new approach to the regulation of termination charges, which are set by means of delegated legislation and which are uniform for the whole of the EU.

The regulation of net neutrality, in turn, can be seen as the expression of an approach that promotes consumers’ interest in a direct manner, that is, not as a side effect of effective competition. The promotion of consumer interest in such a direct way is notable insofar as the Framework is, in fact, expressly biased against dictating the nature and characteristics of the services to be provided by operators.^108^ In accordance with the original design of the regime, intervention was to take place, if at all, at the wholesale level.^109^ The net neutrality regime seems to be based on a new, different logic. It advances its objectives by providing for a right for consumers to access and distribute the content of their choice, from the device of their choice, by means of their Internet access service.^110^ In this sense, not only does it prescribe what is in the end-users’ interest (as opposed to leaving the matter to market forces), but also directly provides for measures to ensure that the desired outcomes are achieved.

The rise of consumer protection and the internal market are not isolated instances of the recalibration of the objectives of the Framework. In fact, a more general trend can be observed across the board. One should note, in particular, that industrial policy considerations became relevant in subsequent iterations of the regime. Under the Code, the first objective of the Framework is now the promotion of ‘connectivity and access to … very high capacity networks’.^111^ In contradiction with the original design, this objective is to be achieved not only by injecting effective competition, but also, in some cases, by reducing the competitive pressure faced by firms. The Code provides for some instances in which the development of infrastructure is prioritised over effective competition. Article 76, in particular, allows firms with significant market power to be subject to lighter regulatory obligations when they agree to a co-investment arrangement with rivals.^112^ In such scenarios, the industrial policy objective takes precedence.

Even though the recalibration of objectives appears to have contributed to the demise of a future-proof approach to regulation, one must emphasise that this outcome was not inevitable. The observed recalibration could have taken place in a manner consistent with the market analysis procedure. Article 76 of the Code provides an example in this sense. While it heralds the rise of industrial policy considerations, it does not depart from the default approach, which demands that significant market power be established prior to the administration of remedies. In fact, the evaluation of the terms of the co-investment decision, and of the consequences that follow, is to be undertaken by regulatory authorities, just like the rest of the core duties imposed under the Framework.

An approach similar to the one enshrined in article 76 would have been conceivable for net neutrality, and roaming and termination charges. In fact, a recalibration of objectives is not only compatible with a future-proof approach to regulation; it is arguably the only sustainable one. Suffice it to mention, in this regard, the foreseeable evolution of the regulation of roaming and termination rates. As this article was under preparation, the roaming regime was being amended with a view to implementing new legislation.^113^ One of the issues that became apparent in the context of the review of the Roaming Regulation is the possible need to introduce a mechanism to phase out intervention as a result of the economic and technological evolution of roaming markets—that is, exactly what the market analysis procedure already provides.^114^ In addition, the review addressed the question of whether intervention need account for developments such as the rise of machine-to-machine services and the Internet of Things).^115^ Five years following the review, it seems clear that some changes may be necessary to ensure that legislation remains future-proof.^116^

### C. The Rise of New Regulatory Philosophies

There is a third reason behind the decline of the future-proof approach to intervention. It is difficult to ignore the fact that the principles and objectives of the regime are the product of a particular regulatory philosophy, which competes with other views about the role of the state in the economy and about the most effective way to deal with uncertainty. Seen from this angle, the Framework is just a manifestation of the preferences of legislatures and other actors at a given point in time. As and when other philosophies gain ground, the principles originally shaping intervention may give way to competing approaches. For the same reason, the aspiration to ensure that intervention is future-proof may not necessarily last long—or may no longer be a priority. Put differently, governments and legislatures may be dynamically inconsistent, but for reasons other than those mentioned above: they may come to question the very premises underpinning the original regime.

The Framework is the product of an approach to intervention that one can term technocratic. Its most salient characteristics are its reliance on expertise and experience, as well as the relative modesty of its ambitions. In line with the principles outlined above, administrative action is typically warranted, under this philosophy, where there is a clear justification (grounded on the expert consensus) for intervention. In this sense, the existence of a market failure is the primary reason for interfering with liberalised markets. In the absence of a compelling reason for action, the regime relies on market outcomes. This modesty reflects a concern with the unintended consequences of regulation and with the distortions it may create. In this sense, the outcomes resulting from the operation of market forces are favoured on balance (whether this is by not taking action absent a market failure or by seeking to mimic effectively competitive segments).

There is no reason to expect that this approach will be systematically favoured in the long run. Governments and legislatures may prove unwilling to accept the structures and outcomes resulting from a philosophy that favours the unfettered operation of markets (even effectively competitive ones) and may seek to interfere with them to advance a particular set of values. There are several reasons why this may be true. For instance, governments and/or legislatures may seek to interfere with markets to redistribute rents across the value chain. Regulation may be used, in other words, to subsidise certain activities at the expense of others. Similarly, intervention may seek to interfere with market structures with a view to reallocating power. For instance, governments and/or legislatures may prefer to disperse power in an industry and ban vertical or conglomerate combinations, even when they would have been efficiency-enhancing.

The successive reviews of the Framework are consistent with the rise of a new understanding of regulation and its relationship with economic and technological realities. These reviews appear to reflect not only a reluctance to accept the outcomes resulting from the choices originally made, but also the very principles underpinning it. Take the example of net neutrality. From an economic perspective, such obligations amount to the subsidisation, by Internet service providers, of content providers.^117^ The redistribution of rents across the value chain through regulation can be rationalised in a number of ways. First, it can be seen as a move that promotes creativity and innovation: content providers would be favoured over network operators. Second, favouring a relatively decentralised ecosystem in which infrastructure providers do not favour, or discriminate against, some content providers (whether affiliated or unaffiliated) could promote new entry and ensure that markets remain contestable.^118^

If anything, the evolution of regulation in the EU suggests that the move heralded by the net neutrality rules introduced in the Framework is not an isolated instance, but rather an indicator of a broader trend. The prevailing attitude towards intervention in the economy appears to be less reliant on expertise, is less deferential to market-based solutions and more willing to tweak and restructure industries so that they conform to the vision that is preferred from a policy-making standpoint. With this understanding of regulation, the ambition of ensuring that regulation is future-proof may become a second- or third-order consideration, if it is considered at all. The Digital Markets Act,^119^ which represents a step back to more traditional approaches to intervention, is a clear example in this sense.

Digital markets (such as search engines, social networks and the online marketplace) are, if anything, more dynamic and more prone to dramatic shifts.^120^ However, the Commission’s vision, advanced in its proposal and embraced by the legislature, is indicative of a more rigid and less tailored approach to regulation than that enshrined in the Framework. The Digital Markets Act relies on a ‘three-criteria’ test that might remind one of the market analysis procedure described above. Any similarities, to the extent that they exist, are superficial.^121^ The purpose of the test under the Framework is to identify, on a market-by-market basis, the levels of the value chain where *ex ante* intervention is necessary to promote and sustain effective competition (and roll back intervention where it is not). The ‘three-criteria’ test under the Digital Markets Act, on the other hand, is to single out firms (as opposed to markets) that have a certain size (but not necessarily a substantial degree of market power, let alone the ability and/or incentive to restrict competition on neighbouring markets).^122^

One of the key features of the Framework, as explained above, is the decoupling between objectives and instruments. This technique ensures that intervention is tailored to the needs of specific markets and that it adjusts over time. The Digital Markets Act, on the other hand, abandons this approach. Instead, regulatory duties are directly enshrined in legislation. Whether the imposition of these duties is or remains necessary to advance the stated objectives of the proposal (namely fairness and contestability^123^) is not a relevant consideration, nor one that demands, in any event, a market-by-market assessment. The necessity of the obligations is just assumed. The flexibility allowed under the proposal concerns the specification of these regulatory duties^124^ and the expansion of the range of obligations to cover other activities.^125^

## 6. Conclusions

The aim of this article was to provide an overview of the evolution of a specific regulatory regime that was specifically designed to be future-proof. The picture that emerges from the analysis is mixed. On the one hand, the Framework has succeeded in its objectives, in the sense that it has proved able to adjust to the economic and technological transformation of the telecommunications industry. The market analysis procedure, as the cornerstone of the regime, has been able to deal with (and will continue to be able to deal with) the erosion of incumbent operators’ market power, the rollout of new (fixed and wireless) infrastructure and a changing competitive landscape. Its decentralised nature has also proved to be a major forum for experimentation by regulatory authorities, which have been able to develop creative and tailored solutions to emerging challenges. At the margin, the Framework did not capture some issues of importance for the dynamics of the industry (in particular, the exploitation of audiovisual content and the rise of OTT services), but such aspects are relatively minor.

On the other hand, every revision of the Framework has represented a move away from the substantive and institutional mechanisms originally put in place to ensure that intervention would remain future-proof. The fundamental feature underpinning the market analysis procedure, which is the decoupling of the objectives of the regime (enshrined in legislation) and the instruments to implement those same objectives (left to the regulatory authorities), has progressively been abandoned. The two most significant developments in the field, which are the elimination of roaming tariffs and net neutrality, depart from that approach and directly provide for remedies in legislation—and this without evaluating whether the preconditions for intervention are fulfilled. In this sense, the evolution of the regime reveals a problem of intertemporal consistency on the part of the legislature.

This analysis hints at two main lessons for regulation in general. The first is that the success of future-proof regulation does not depend—at least, not primarily—on the *ex ante* design of a regime, but on the ability of authorities and legislatures to credibly commit, over time, to the same design. The challenge, in other words, is fundamentally exogenous, as opposed to endogenous. Against this background, it is submitted that any realistic attempt to design a lasting future-proof regime will have to incorporate mechanisms that make it costly for stakeholders, in particular legislatures and authorities, to depart from the approach enshrined in it. In a similar vein, it may be necessary to develop procedures in which the trade-offs and expected benefits from intervention (and, by the same token, the costs of departing from the principles, techniques and objectives of the regime) are regularly evaluated and highlighted.

A second, more controversial and far-reaching conclusion is that the idea of future-proof regulation might be inherently flawed, or premised on an unrealistic, if not contradictory, ambition. To some extent, it appears to assume that the world around regulation is subject to constant change, but that somehow the permanent mutation of economic and technological realities will fail to influence how legislatures define their priorities and their views on regulation and its role. The examples discussed in this article show that even the recalibration of the objectives and/or of perceptions of risk may go so far as to lead to the abandonment of the original logic of a regime. Thus, the ambition of future-proof regulation may be downplayed or might not be prioritised over other considerations. And the very problems that future-proof regimes seek to address (namely regulatory distortions and rigid legislation) may prove more intractable than previously assumed.

## Footnotes

1 In this sense, see Sofia Ranchordás and Yaniv Roznai (eds), *Time, Law, and Change: An Interdisciplinary Study* (Hart Publishing 2020).

2 For an analysis of the concept, see Sofia Ranchordás and Mattis van’t Schip, ‘Future-Proofing Legislation for the Digital Age’ in Ranchordás and Roznai (n 1). See also European Economic and Social Committee, *Future Proof Legislation* (SC/045, 2016).

3 See in particular Directive (EU) 2018/1972 of the European Parliament and the Council of 11 December 2018 establishing the European Electronic Communications Code [2018] OJ L321/36 (Electronic Communications Code). The UK regime continues to apply, in essence, the same approach, The Electronic Communications and Wireless Telegraphy (Amendment etc) (EU Exit) Regulations 2019.

4 For an analysis of the concept and its implications, see Pablo Ibáñez Colomo, *European Communications Law and Technological Convergence: Deregulation, Re-regulation and Regulatory Convergence in Television and Telecommunications* (Kluwer 2011).

5 See European Commission, ‘Explanatory Note accompanying the Commission Recommendation on relevant product and service markets within the electronic communications sector susceptible to ex ante regulation in accordance with Directive (EU) 2018/1972 of the European Parliament and of the Council of 11 December 2018 establishing the European Electronic Communications Code’ SWD (2020) 337 final.

6 Ibáñez Colomo (n 4).

7 Martin Peitz, Heike Schweitzer and Tommaso Valletti, ‘Market Definition, Market Power and Regulatory Interaction in Electronic Communications Markets’ CERRE Study (Brussels, 29 October 2014).

8 Commission Directive 96/19/EC of 13 March 1996 amending Directive 90/388/EEC with regard to the implementation of full competition in telecommunications markets [1996] OJ L74/13.

9 For an analysis of the process, see Pierre Larouche, *Competition Law and Regulation in European Telecommunications* (Hart Publishing 2000).

10 European Commission, ‘Green Paper on the Development of the Common Market for Telecommunications Services and Equipment’ COM (87) 290 final.

11 European Commission, ‘Towards a new framework for Electronic Communications infrastructure and associated services—The 1999 Communications Review’ COM (1999) 539 final.

12 European Commission, ‘Green Paper on the convergence of the telecommunications, media and information technology sectors, and the implications for Regulation—Towards an information society approach’ COM (97) 623.

13 ibid 22.

14 ibid 5.

15 For a non-technical description, see Jonathan E Nuechterlein and Philip J Weiser, *Digital Crossroads: American Telecommunications Policy in the Internet Age* (2nd edn, MIT Press 2013).

16 A concern identified in Larouche (n 9).

17 1999 Review (n 11) 14–15.

18 On the concept of technology neutrality, see Ilse van der Haar, *The Principle of Technological Neutrality—Connecting EC Network and Content Regulation* (Tilburg University 2008).

19 See the original definition (subsequently changed) in the Directive 2002/21/EC of the European Parliament and of the Council of 7 March 2002 on a common regulatory framework for electronic communications networks and services [2002] OJ L108/33 (Framework Directive). In art 2(a) of the Directive (no longer in force), an ‘electronic communications network’ was defined as meaning ‘transmission systems and, where applicable, switching or routing equipment and other resources which permit the conveyance of signals by wire, by radio, by optical or by other electromagnetic means, including satellite networks, fixed (circuit- and packet-switched, including Internet) and mobile terrestrial networks, electricity cable systems, to the extent that they are used for the purpose of transmitting signals, networks used for radio and television broadcasting, and cable television networks, irrespective of the type of information conveyed’.

20 ibid art 2(c), which defined an ‘electronic communications service’ as ‘a service normally provided for remuneration which consists wholly or mainly in the conveyance of signals on electronic communications networks, including telecommunications services and transmission services in networks used for broadcasting, but exclude services providing, or exercising editorial control over, content transmitted using electronic communications networks and services; it does not include information society services, as defined in Article 1 of Directive 98/34/EC, which do not consist wholly or mainly in the conveyance of signals on electronic communications networks’.

21 1999 Review (n 11) 21.

22 ibid.

23 Framework Directive (n 19) Recital 5.

24 ibid. See in particular Directive 2010/13/EU of the European Parliament and of the Council of 10 March 2010 on the coordination of certain provisions laid down by law, regulation or administrative action in Member States concerning the provision of audiovisual media services (Audiovisual Media Services Directive) [2010] OJ L95/1.

25 The Electronic Communications Code (n 3) defines the universal service, in Recital 212, as ‘a safety net to ensure that a set of at least the minimum services is available to all end-users and at an affordable price to consumers, where a risk of social exclusion arising from the lack of such access prevents citizens from full social and economic participation in society’.

26 Framework Directive (n 19) arts 8(2)–8(4).

27 Electronic Communications Code (n 3). In the previous incarnation, the Framework Directive (n 19) introduced the market analysis procedure in arts 14–16.

28 art 67(5)(a) of the Electronic Communications Code (n 3).

29 European Commission, ‘Guidelines on market analysis and the assessment of significant market power under the EU regulatory framework for electronic communications networks and services’ SWD (2018) 124 final.

30 art 63 of the Electronic Communications Code (n 3).

31 Case 85/76 *Hoffmann-La Roche & Co AG v Commission* ECLI:EU:C:1979:36, para 38.

32 SMP Guidelines (n 29).

33 art 63 of the Electronic Communications Code (n 3); *Hoffmann-La Roche* (n 31).

34 art 67(1) of the Electronic Communications Code (n 3) codifies the ‘three-criteria’ test, which was previously found in the Recommendations on the definition of relevant markets issued by the Commission.

35 ibid art 64(1).

36 ibid art 67(1); Commission Recommendation of 18 December 2020 on relevant product and service markets within the electronic communications sector susceptible to ex ante regulation in accordance with Directive (EU) 2018/1972 of the European Parliament and of the Council of 11 December 2018 establishing the European Electronic Communications Code [2020] OJ L439/23, para 22.

37 ibid para 19.

38 art 67(2) of the Electronic Communications Code (n 3).

39 See in particular ibid art 77.

40 ibid art 69.

41 ibid art 70.

42 ibid art 71.

43 ibid art 77.

44 ibid art 78.

45 These obligations are typically found in regulatory regimes. In this sense, see Martin Cave, Robert Baldwin and Martin Lodge, *Understanding Regulation* (2nd edn, OUP 2011) ch 23.

46 This outcome is consistent with the ‘ladder of investment’ theory, whereby new entrants would progressively invest in new infrastructure. In this sense, see Martin Cave, ‘Encouraging Infrastructure Competition via the Ladder of Investment’ (2006) 30 Telecommunications Policy 223.

47 On experimentation in the EU outside the formal scope of legislation, see Charles F Sabel Jonathan Zeitlin, ‘Experimentalist Governance’ in David Levi-Faur (ed), *The Oxford Handbook of Governance* (OUP 2012).

48 Commission Recommendation of 11 February 2003 on relevant product and service markets within the electronic communications sector susceptible to ex ante regulation in accordance with Directive 2002/21/EC of the European Parliament and of the Council on a common regulatory framework for electronic communication networks and services [2003] OJ L114/45.

49 Commission Recommendation of 17 December 2007 on relevant product and service markets within the electronic communications sector susceptible to ex ante regulation in accordance with Directive 2002/21/EC of the European Parliament and of the Council on a common regulatory framework for electronic communications networks and services [2007] OJ L344/65.

50 Commission Recommendation of 9 October 2014 on relevant product and service markets within the electronic communications sector susceptible to ex ante regulation in accordance with Directive 2002/21/EC of the European Parliament and of the Council on a common regulatory framework for electronic communications networks and services [2014] OJ L295/79.

51 2020 Recommendation (n 36).

52 For an extensive discussion, see the Explanatory Note accompanying the 2020 Recommendation (n 5).

53 ibid 49–51.

54 ibid: ‘given the small number and often limited geographic reach of competitors operating their own alternative infrastructure it is unlikely that without continued regulatory intervention, the competitive dynamics in this market will change significantly on a national scale over the foreseeable future’.

55 See in particular art 79 of the Electronic Communications Code (n 3).

56 Under the concept of functional separation, the incumbent’s infrastructure is separated from the rest of its activities and placed in an independent business. In this sense, see Martin Cave, ‘Six Degrees of Separation: Operational Separation as a Remedy in European Telecommunications Regulation’ (2006) 64 Communications & Strategies 89.

57 art 72 of the Electronic Communications Code (n 3).

58 Commission communication concerning the review under competition rules of the joint provision of telecommunications and cable TV networks by a single operator and the abolition of restrictions on the provision of cable TV capacity over telecommunications networks [1998] OJ C71/4. For an overview of the market structure in the different Member States, see European Commission, ‘Digital Economy and Society Index (DESI) 2020’.

59 Commission Recommendation of 20 September 2010 on regulated access to Next Generation Access Networks (NGA) [2010] OJ L251/35.

60 Explanatory Note accompanying the 2020 Recommendation (n 5).

61 2020 Recommendation (n 36) para 19. On this point, see in particular Case C-424/07 *Commission v Germany* ECLI:EU:C:2009:749.

62 ibid.

63 In this sense, see arts 73 and 74 of the Electronic Communications Code (n 3).

64 Case C-518/11 *UPC Nederland BV v Gemeente Hilversum* ECLI:EU:C:2013:709.

65 ibid para 41.

66 See eg Mark Sweney, ‘BT Sport Keeps Champions League Rights in £1.2bn Deal for 2021–24’ *The Guardian* (London, 15 November 2019).

67 See eg *Telefónica/DTS* (Case C/0612/14) CNMC Decision of 22 April 2015.

68 ibid.

69 In fact, the ‘three-criteria’ test, mentioned above, assumes the limits of competition law. The third limb of the test makes an explicit reference to the ability of competition law to handle complex remedies.

70 For an extensive discussion in this sense, see Niamh Dunne, *Competition Law and Economic Regulation: Making and Managing Markets* (CUP 2015).

71 Ofcom, ‘Pay TV Statement’ (31 March 2010).

72 See Peitz, Schweitzer and Valletti (n 7).

73 See art 61 of the Electronic Communications Code (n 3).

74 See Peitz, Schweitzer and Valletti (n 7).

75 Electronic Communications Code (n 3) art 2(6).

76 ibid art 2(7).

77 ibid art 61.

78 Regulation (EU) 2015/2120 of the European Parliament and of the Council of 25 November 2015 laying down measures concerning open internet access and amending Directive 2002/22/EC on universal service and users’ rights relating to electronic communications networks and services and Regulation (EU) No 531/2012 on roaming on public mobile communications networks within the Union [2015] OJ L310/1.

79 The original piece of legislation (no longer in force) is Regulation (EC) No 717/2007 of the European Parliament and of the Council of 27 June 2007 on roaming on public mobile telephone networks within the Community and amending Directive 2002/21/EC [2007] OJ L171/32.

80 Ian Forrester, ‘Sector-Specific Price Regulation or Antitrust Regulation: A Plague on Both Your Houses?’ in Claus-Dieter Ehlermann and Mel Marquis (eds), *European Competition Law Annual 2007: A Reformed Approach to Article 82 EC* (Hart Publishing 2008).

81 ibid.

82 Regulation 2015/2120 (n 15).

83 For a discussion of the concept of net neutrality, see Nuechterlein and Weister (n 15); in particular, see Lucie C Audibert and Andrew D Murray, ‘A Principled Approach to Network Neutrality’ (2016) 13 SCRIPTed 118.

84 ibid.

85 These practices are all mentioned in Regulation 2015/2120 (n 15).

86 This is how the issue was originally understood: in this sense, see Filomena Chirico, Ilse van der Haar and Pierre Larouche, ‘Network Neutrality in the EU’ (2007) TILEC Discussion Paper No 2007-030.

87 ibid.

88 In this sense, see Neelie Kroes, ‘Vice-President Kroes to Propose Action on Consumer Choice and “Net Neutrality”’ MEMO/12/389.

89 Directive 2009/136/EC of 25 November 2009 amending Directive 2002/22/EC on universal service and users’ rights relating to electronic communications networks and services, Directive 2002/58/EC concerning the processing of personal data and the protection of privacy in the electronic communications sector and Regulation (EC) No 2006/2004 on cooperation between national authorities responsible for the enforcement of consumer protection laws [2009] OJ L337/11.

90 2003 Recommendation (n 48).

91 For a theoretical background, see Jean-Jacques Laffont and Jean Tirole, *Competition in Telecommunications* (MIT Press 1999).

92 See eg *Wholesale call termination on individual public telephone networks at a fixed location), 1669 Wholesale voice call termination on individual mobile networks* (Case FR/2014/1668); *Wholesale voice call termination on individual mobile networks* (Case FR/2014/1669) () and *Wholesale SMS termination on individual*
*mobile networks* (Case FR/2014/1670); Explanatory Note accompanying the 2020 Recommendation (n 5).

93 Commission Delegated Regulation (EU) 2021/654 of 18 December 2020 supplementing Directive (EU) 2018/1972 of the European Parliament and of the Council by setting a single maximum Union-wide mobile voice termination rate and a single maximum Union-wide fixed voice termination rate [2021] OJ L137/1.

94 This concept is borrowed from economics, where it has been widely used and its origins traced back to Finn E Kydland and Edward C Prescott, ‘Rules Rather than Discretion: The Inconsistency of Optimal Plans’ (1977) 85 Journal of Political Economy 473.

95 For a discussion of these two dimensions of competition, see Carl Shapiro, ‘Competition and Innovation: Did Arrow Hit the Bull’s Eye?’ in J Lerner and S Stern (eds), *The Rate and Direction of Inventive Activity Revisited* (University of Chicago Press 2012); Pablo Ibáñez Colomo, ‘Restrictions on Innovation in EU Competition Law’ (2016) 41 EL Rev 201.

96 Forrester (n 80).

97 ibid.

98 In the context of network industries regulation, it is well accepted that more vulnerable consumers are less likely to benefit from the opportunities. See, by analogy, Competition and Markets Authority, *Energy Market Investigation—Final Report*, paras 8.147–8.152.

99 ‘Zero-rating’ refers to instances in which some services are favoured insofar as they do not count towards the monthly data allowance and thus can be consumed without limits. In this sense, see BEREC Guidelines on the Implementation by National Regulators of European Net Neutrality Rules, BoR (16) 127. See also Joined Cases C-854/19, C-5/20 and C-34/20 *Vodafone GmbH v Bundesrepublik Deutschland* ECLI:EU:C:2021:675.

100 A fast lane would allow a particular service to be treated with priority when transported through the network.

101 Guidance on the Commission’s enforcement priorities in applying Article 82 of the EC Treaty to abusive exclusionary conduct by dominant undertakings [2009] OJ C45/7, para 34; Guidelines on the assessment of non-horizontal mergers under the Council Regulation on the control of concentrations between undertakings [2008] OJ C256/6, paras 13–14.

102 ibid.

103 See n 99.

104 C Scott Hemphill, ‘Network Neutrality and the False Promise of Zero-Price Regulation’ (2008) 25 Yale Journal on Regulation 135.

105 Such was, essentially, the outcome following the revision of the Framework in 2009. The Netherlands was the first country to react, after the controversy that followed the behaviour of the incumbent operator. In this sense, see ‘Netherlands Makes Net Neutrality A Law’ (BBC News, 23 June 2011).

106 art 8 of the Framework Directive (n 19).

107 arts 84–92 of the Electronic Communications Code (n 3).

108 ibid art 83.

109 2020 Recommendation (n 36).

110 art 3(1) of Regulation 2015/2120 (n 15).

111 art 3 of the Electronic Communications Code (n 3).

112 BEREC Guidelines to foster the consistent application of the conditions and criteria for assessing co-investments in new very high capacity network elements (Article 76 (1) and Annex IV EECC), BoR (20) 232.

113 The text eventually adopted is Regulation (EU) 2022/612 of the European Parliament and of the Council of 6 April 2022 on roaming on public mobile communications networks within the Union (recast) [2022] OJ L115/1 (Roaming Regulation). Luca Bertuzzi, ‘EU Renews Roaming until 2032 with Gradual Reductions for Data Cost’ *Euractiv* (Brussels, 9 December 2021).

114 European Commission, ‘Summary Report on the public consultation on the review and prolongation of the Roaming Regulation 2020’.

115 ibid.

116 There is, in fact, an explicit reference to the concept in the legislative proposal for a new roaming regulation. In this sense, see Roaming Regulation (n 113) 3.

117 Robin S Lee and Tim Wu, ‘Subsidizing Creativity through Network Design: Zero-Pricing and Net Neutrality’ (2009) 23 Journal of Economic Perspectives 61.

118 In this sense, see Barbara van Schewick, *Internet Architecture and Innovation* (MIT Press 2010).

119 Commission, ‘Proposal for a Regulation of the European Parliament and of the Council on contestable and fair markets in the digital sector (Digital Markets Act)’ COM (2020) 842 final. The version agreed by the EU institutions following the legislative process can be found at www.consilium.europa.eu/media/56086/st08722-xx22.pdf accessed 8 July 2022. This version keeps the essential features of the Proposal discussed in this article.

120 For an in-depth discussion of the features of digital markets, see Nicolas Petit, *Big Tech and the Digital Economy: The Moligopoly Scenario* (OUP 2020).

121 Pablo Ibáñez Colomo, ‘The Draft Digital Markets Act: A Legal and Institutional Analysis’ (2021) 12 Journal of European Competition Law & Practice 561.

122 art 3 of the Proposal for a Digital Markets Act (n 118).

123 ibid Recitals 28 and 32.

124 ibid art 6.

125 ibid art 10.

## Notes

I am grateful to the participants in the LSE Staff Seminar and the Jean Monnet Research Workshop at which I presented an earlier draft of this article. In accordance with the ASCOLA Declaration of Ethics, I am happy to clarify that I have nothing to disclose.

